# Chinese version of *Dominic Interactive* – A self-report video game for assessing mental health in young children

**DOI:** 10.3389/fpsyt.2023.1149970

**Published:** 2023-04-24

**Authors:** Viviane Kovess-Masfety, Guoli Yan, Huifang Yin, Ling Sun, Xiaofei Hou, Minghui Li, Peiyao Li, Xuyang Su, Michael R. Phillips, Guangming Xu

**Affiliations:** ^1^Tianjin Anding Hospital, Mental Health Center of Tianjin Medical University, Tianjin, China; ^2^LPPS, University of Paris, Paris, France; ^3^Suicide Research and Prevention Center, Shanghai Mental Health Center, Shanghai Jiao Tong University School of Medicine, Shanghai, China; ^4^Department of Psychiatry and Epidemiology, Columbia University, New York, NY, United States

**Keywords:** test, video game, survey, children, psychiatric diagnoses, China, screening

## Abstract

**Objectives:**

Assess the validity of the Chinese version of the *Dominic Interactive* (DI), a 91-item, video-based diagnostic screening instrument for children that assesses four internalized disorders (phobias, separation anxiety disorder, generalized anxiety disorder, and major depressive disorder) and three externalized disorders (attention-deficit/hyperactivity disorder, conduct disorder, oppositional defiant disorder).

**Methods:**

(1) Compare DI-generated “probable” or “possible” diagnoses to diagnoses based on the Development and Well-Being Assessment (DAWBA) instrument in 113 psychiatric outpatients and 20 community controls. (2) Administer DI to 1,479 children from elementary schools in Tianjin.

**Results:**

In the validation sample, DI with DAWBA concordance was much greater for internalized disorders (mean Kappa = 0.56) than for externalized disorders (mean kappa = 0.11). The positive predictive value of DI diagnoses ranged from 0.96 (generalized anxiety disorder) to 25% (oppositional defiant disorder) and negative from 0.81 to 0.96. Using “probable” cuts provides better results. In the survey, prevalence of probable DI disorders ranged from 1.0% (conduct disorder) to 13.1% (phobias). Internal consistency of all DI items was excellent (Cronbach alpha = 0.93) and that of the seven subscales ranged from 0.64 (phobias) to 0.87 (major depressive disorder). In multilevel SEM analyses, SRMR (Standardized root mean square residual) or each of the seven diagnoses was below 0.08 and each coefficient of determination was below 0.60.

**Conclusion:**

The Chinese DI is a convenient method of screening common mental disorders in Chinese children mainly for internalized disorders, which are the most prevalent diagnoses in that population. However its high negative predictive values for externalized could be used for screening.

## 1. Introduction

Several self-completion survey instruments have been used in China to assess the mental health of children and adolescents (1–12). Most of these instruments are suitable for children 10 years of age or older. When used in children younger than this, these instruments are typically read to the child by a parent, teacher, or another adult; in these cases, it is not known whether the child understands the items in the scales, so the validity of using this method to assess the mental health status of young children in China is uncertain. Nevertheless, previous research in Western countries has demonstrated that children as young as 5 or 6 can provide valid self-reports of their mood and feelings if the assessment methods are specifically tailored for young children (13–16).

One such self-report method, the video-based *Dominic Interactive* (DI), has been validated and successfully employed in surveys of primary school children 6 to 12 years of age in French communities, English communities, and indigenous communities in Canada (17–19), and in the United States and Brazil (20), France (21), Spain (22), the Netherlands (23), and in European multi-country projects (24). The DI video assesses children’s mental health symptoms by eliciting their responses to depictions of cartoon-like situations on a computer, tablet, or cell phone. The cartoon’s main character is adjusted to match the respondent’s gender and ethnicity, increasing the likelihood that the child will identify with the character. The DI starts with a short tutorial to ensure that the child understands how to select “yes” or “no” to answer the 91 questions included in the DI package. The questions appear in written form on the screen and are simultaneously read aloud as part of the video program. The children must complete one question before proceeding to the next question, but they can interrupt the video and resume at the same location later. The presented situations were designed to depict the emotional and behavioral symptoms of seven mental disorders most commonly present at these ages such as described in the Diagnostic and Statistical Manual of Mental Disorders version V (25): attention-deficit/ hyperactivity disorder (ADHD), conduct disorder (CD), oppositional defiant disorder (ODD), phobias (PH), separation anxiety disorder (SAD), generalized anxiety disorder (GAD), and major depressive disorder (MDD). The presence or absence of nine specific phobias considered by DI questions were subclassified into four types: animal type (insects, dogs, cat, and spiders), environmental type (storms, heights), situational type (elevators, hallways), or other types (persons in costumes) (26). Ten of the 91 DI questions are neutral items not associated with any of the seven diagnoses.

For each disorder, DI categorizes the diagnosis as “possible” if the number of reported symptoms is one standard deviation above the mean number reported by a standard sample and as “probable” if the number of reported symptoms is two standard deviations or more above the mean reported by the standard sample (19, 27). The standard sample used to select these means and standard deviations included 308 French-speaking and 277 English-speaking children living in Montreal (Canada), including 453 (75%) from the general community and 132 (25%) from outpatient child psychiatric clinics.

The objectives of the paper are (1) to adapt the DI to Chinese; (2) to examine evidence of validity of the Chinese version of DI; and (3) to analyze the properties of DI in a school community sample of primary school children in Tianjin.

## 2. Materials and methods

### 2.1. Development of the Chinese version of *Dominic Interactive*

Multiple versions of DI make it suitable for use with girls and boys from different racial and linguistic groups. The first step in completing DI asks the child to select his/her gender and his/her appearance (Caucasian, African, Asian, or Arabic); the child then selects their preferred language.

The current project developed the Mandarin Chinese version of the DI: in order to fit the Chinese culture, the child was named “Xiaoming”; a frequent given name which could fit both genders. Each of the 91 DI questions was translated into Mandarin by a bilingual native Chinese professional translator in Canada and revised after back-translation by another bilingual speaker. The Tianjin investigators considered the translated questions suitable for use with children in Tianjin but recommended modifying two of the DI slides. The slides illustrating feelings of guilt and self-blame (part of the assessment of depression) that show a child being rejected by a parent followed by a slide of the crying child leaving home with a suitcase was not considered plausible in China, so they were replaced by a slide showing the child failing at school followed by a slide of the crying child back at home being blamed by parents.

**Figure 1:**
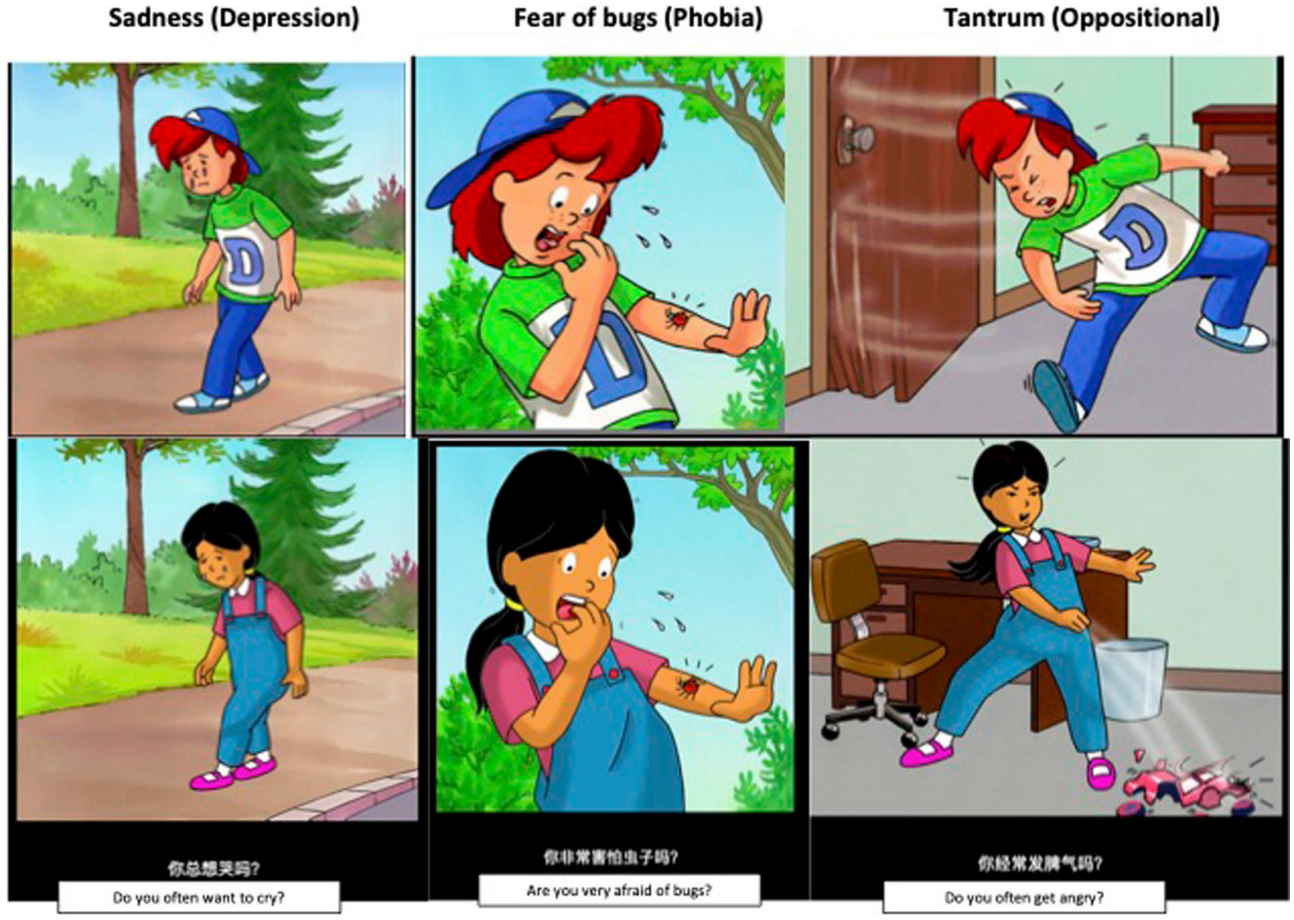
Examples of *Dominic Interactive* slides presented to Caucasian boys (top row) and Asian girls (bottom row).

Finally, a native Mandarin speaker recorded the audio recording that accompanied the Chinese version of the DI video presentation. A pilot survey with 12 participants (2 from each grade, from Grade 1 to Grade 6) in Tianjin confirmed that target respondents could understand the content. Examples of the presentation are shown in Figure 1.

### 2.2. Validation of the Chinese version of *Dominic Interactive* versus DAWBA

To validate the Chinese version of DI, we compared the possible and probable diagnoses generated by the Chinese DI to diagnoses determined by the administration of the Chinese version of the Development and Well-Being Assessment (DAWBA) by three child psychiatrists in a sample of children that included both psychiatric outpatients and community controls.

The DAWBA (28) is a structured computerized interview designed to assess DSM-V (25) diagnoses in 5- to 17-year-old children and adolescents. Sept of the diagnoses considered by DAWBA were assessed in this study: separation anxiety, specific phobia, generalized anxiety disorder, major depression, ADHD/hyperkinesis, conduct and oppositional-defiant disorder. Previous reports indicate that DAWBA diagnoses have high inter-rater reliability and that the DAWBA effectively differentiates clinical and non-clinical cases (29). The Chinese version of DAWBA, which has shown satisfactory validity and reliability (30), has been used in many studies in China, including a large study of mental disorders in children living in Northeast China (31).

In this validation study, trained child psychiatrists administered the DAWBA in-person to children’s parents (primarily mothers) and conducted separate DAWBA interviews by phone with the children’s teachers. The algorithm available at www.dawba.com took into consideration the parent and teacher interview data and then a third-party child psychiatrist reviewed all the information and determined whether there is a positive or negative diagnosis.

All children 6 to 13 years of age who attended the pediatric outpatient clinic of Tianjin Anding Psychiatric Hospital from November 2020 to August 2021 were potential subjects, excluding those with autism, child psychosis, or intellectual development problems. When each child attended the clinic, a psychiatrist introduced the study procedures to the child’s parents and asked for their consent. The parents of 120 of the 146 (82.2%) potentially eligible children signed the study consent form, but 2 of them were unable to complete DAWBA due to time constraints and 4 of the children did not complete DI due to reported discomfort during the test. Among the consenting parents, 100 agreed to solicit the participation of their child’s teacher, but 20 of the teachers refused to participate. This resulted in 114 (95%) completed interviews with parent’s DAWBA and the child’s DI, 80 of which had an accompanying DAWBA completed by the child’s teacher; one case has to be deleted since he was too old to be included. Twenty other children without reported mental health problems were identified by research staff and assessed using the same method (i.e., DAWBA was administered in-person to a parent and *via* telephone to the teacher, and the DI was completed by the child). The total combined sample of 133 children in this validation study included 68 boys and 65 girls; the mean age was 10.7 (S.D. = 1.6) years.

A website link to the Chinese version of DI developed by the research team was provided by the original author of the DI. After the parent consented, each child was taken to a private room in the outpatient clinic by an investigator to complete the DI. Before starting the test, the investigator helped the child open the links and taught the child how to respond to the questions.

### 2.3. Survey of primary school children in Tianjin using the Chinese version of *Dominic Interactive*

The Dongli District of Tianjin (population, 445,500) was selected for the survey because (1) it included urban, suburban, and rural communities; (2) its sociodemographic characteristics were reasonably representative of Tianjin’s overall characteristics; and (3) the Tianjin Anding Hospital’s research staff had previously collaborated with the district’s school authorities. The 36 primary schools in Dongli District of Tianjin were subclassified by their location into 12 rural, 15 suburban, and 9 urban schools. Then one school of each type was randomly selected for participation in the survey.

In October 2020, the three school’s directors were contacted to obtain authorisation for the survey. The head teachers of every class in the schools were informed about survey procedures. These teachers identified 2083 potential students and sent messages to their parents *via* “We chat” (the most widely used social media platform in China) to inform them about the survey and ask them to sign the consent form (that their child brought home) and have the child bring it back to the teacher. In total, 2015 (96.7%) of the invited parents provided their consent.

Every student whose parent consented was given a unique identification number. The parent was then given a letter (carried home by the student) asking them to complete a questionnaire, attach a sticker with the student’s identification number to the questionnaire, enclose the completed questionnaire in a provided envelope (and to seal the envelope), and ask the student to return the envelope to the teacher. The questionnaire completed by the parent included nine sections: information about the structure of the household in which the child is living, about their child’s activities and physical problems, about their attitudes and perception of their child’s mental health, and about their own marital status, education, occupation, ethnicity, economic situation, and personal mental health (including alcohol and tobacco consumption).

Each student was assigned a unique link that could be used to complete the web-based DI at home or at school. At the time of the December 2020 winter vacation, teachers sent the link and guidelines for managing the test to parents *via* “We chat” instructing them how to help the student open the test link (using a computer, tablet, or cell phone), enter their gender and age and complete the DI on their own (informing them that they could terminate the questionnaire at any time if they felt uncomfortable). A total of 593 students completed DI at home. In April 2021, students who did not complete the survey at home were invited to complete it at school: investigators brought laptops to school to complete the test, helped the students open their unique links and complete the background information, and then left the room to allow the students to complete the DI on their own. In total 886 students completed the survey at school. Thus, among the 2015 students whose parents consented, 1,479 (72.1%) successfully completed the DI; the successful completion rate was 86.5% in the rural school and 98.1% in the suburban school, but only 31.3% in the urban school (where the survey had to be prematurely stopped after a single parent complained about the survey to school authorities).

The mean time to complete the DI was 18 min.

### 2.4. Statistical analyses

For the purposes of this analysis, “internalized” disorders included specific phobia, generalized anxiety, separation anxiety, depressive disorder, and “externalized” disorders included oppositional defiant disorder, conduct disorder, and attention-deficit/hyperactivity disorder.

For each of the seven diagnoses considered by DI, we compared the DI diagnostic classifications, using the DI proposed cut points, with the “gold standard” DAWBA clinical assessment. We calculated sensitivity, specificity, positive predictive value, and negative predictive value using both the values for “probable” DI diagnosis and, separately, for the “possible” DI diagnosis. The chance-adjusted agreement of the DI and DAWBA diagnoses were assessed using the Kappa statistic, and the area under the receiving operator characteristic (AUROC) was used to estimate the capacity of the DI to discriminate between the presence and absence of each DAWBA diagnosis. The internal consistency of all 81 DI items associated with diagnoses and of the items in each of the seven DI subscales (one for each type of mental health problem considered) were assessed using Cronbach’s alpha.

The prevalence of the “probable” and “possible” cases of the seven types of mental health problems assessed by the DI identified in the school survey were compared between boys and girls using Pearson chi-square tests. Factors associated with the most prevalent DI diagnosis (phobias) and of any DI diagnosis are assessed using logistic regression analysis.

We used the structural equation modeling (SEM) approach proposed by Kuijpers (32) to assess the results of a seven-factor categorical confirmatory factor analysis based on the item scores of the seven diagnoses assessed by the DI. However, as Kuijpers reported, 9 of the 81 symptom-related items load on more than one subscale (e.g., “irritability” belongs to the GAD, MDD, and ODD subscales), hampering a simple confirmative factor analysis; in the confirmative factor analysis of all 81 items we allocated these items to a single subscale – the subscale for which the items were problem-specific (19) (2 to the MDD subscale, 3 to the ODD subscale, and 4 to the ADHD subscale). We repeated the same SEM for each of the subscales, using all items that make up each subscale (including those that are also used in other subscales). Since the sample was collected into three schools, we used a multilevel command (“cluster” in stata) to compute the SEM providing SRMR (Standardized root mean square residual) and Coefficient of determination.

All analyses were conducted using Stata/IC 15.1 for Mac. *p*-values below 0.05 were considered statistically significant.

## 3. Results

### 3.1. Concordance of the Chinese DI diagnosis with the Chinese DAWBA diagnosis

Compared to the “gold standard” DAWBA diagnoses, the positive predictive value of the “probable” DI diagnosis classification for the seven diagnoses considered – that is, the likelihood that an individual with a DI “probable case” classification of a disorder has the disorder – was very contrasted, from a high agreement for internalized disorders: 0.90 on average to a rather low agreement for externalized disorder: 0.44 on average with the exception for ADHD: 0.67 (Table 1). On the contrary, the corresponding negative predictive value – that is, the likelihood that an individual without the “probable case” DI classification for a disorder does not have the disorder – was quite good for externalized disorders: 0.72 (ranging from 0.92 to 0.79) and fair for internalized disorders: 0.67 (ranging from 0.89 to 0.76).

**Table 1:** Comparison of the diagnostic results of the self-report *Dominic Interactive* (DI) video game (probable cases) to that of the diagnostic result of the clinician-administered Development and Well-Being Assessment (DAWBA) in 133 children in Tianjin, China.

Disorder	Probable DI cases	DAWBA cases	DI/DAWBA results				Sensitivity	Specificity	Positive predictive value (PPV) (25)	Negative predictive value (NPV)	Area under the curve (AOC), (95% CI)	Kappa (SE)	*p*-value
			+/+	+/−	−/+	−/−							
Attention deficit/hyperactivity disorder	32	9	6	3	26	98	0.19	0.97	0.67	0.79	0.58 (0.51–0.65)	0.21 (0.07)	**0.001**
Conduct disorder	8	13	3	5	10	115	0.23	0.96	0.38	0.92	0.59 (0.47–0.72)	0.23 (0.08)	**0.0032**
Oppositional defiant disorder	12	20	3	9	17	104	0.15	0.92	0.25	0.86	0.54 (0.45–0.62)	0.08 (0.09)	0.156
Major depressive disorder	35	47	30	5	17	81	0.64	0.94	0.86	0.83	0.79 (0.72–0.86)	0.62 (0.08)	**<0.0001**
Generalized anxiety disorder	25	50	24	1	26	82	0.48	0.99	0.96	0.76	0.74 (0.66–0.80)	0.52 (0.08)	**<0.0001**
Separation-anxiety disorder	19	27	14	5	13	106	0.52	0.95	0.74	0.89	0.74 (0.64–0.83)	0.53 (0.08)	**<0.0001**
Phobias	35	34	24	11	10	88	0.71	0.89	0.71	0.89	0.80 (0.71–0.88)	0.59 (0.09)	**<0.0001**
Any externalised^1^	18	40	8	10	32	83	20	89	0.44	0.72	0.55 (0.48–0 0.62)	0.11(0.08)	**0.0764**
Any internalised disorder^2^	60	78	54	6	24	49	0.69	0.89	0.90	0.67	0.79 (0.73–0.86)	0.56 (0.08)	**<0.0001**
Any disorder	65	104	63	2	41	27	0.61	0.93	0.97	0.40	0.77 (0.70–0.83)	0.36 (0.07)	**<0.0001**

1 Includes attention-deficit/hyperactivity disorder, conduct disorder, and oppositional defiant disorder.

2 Includes major depressive disorder, generalised anxiety disorder, separation-anxiety disorder, and phobias.

The bold value means *p* < 0.05.

Based on the kappa statistic, the chance-adjusted concordance of probable DI diagnoses with DAWBA diagnoses (Table 1) was much greater for internalized disorders (major depressive disorder, generalized anxiety disorder, separation-anxiety disorder, and phobias) from 0.62 to 0.52 than for externalized disorders (attention-deficit/hyperactivity disorder, conduct disorder, and oppositional defiant disorder) from 0.08 to 0.23. We repeated the same analyses using the “possible “cases (Table 2) which did not produce better results on the contrary.

**Table 2:** Comparison of the diagnostic results of the self-report *Dominic Interactive* (DI) video game [possible cases (including probable cases)] to that of the diagnostic result of the clinician-administered Development and Well-Being Assessment (DAWBA) in 133 children in Tianjin, China.

Disorder	Possible DI cases	DAWBA cases	DI/DAWBA results				Sensitivity	Specificity	Positive predictive value (PPV)	Negative predictive value (NPV)	Area under the curve (AOC) (95% CI)	Kappa (SE)	p-value
			+/+	+/−	−/+	−/−							
Attention deficit/hyperactivity disorder	26	32	12	14	20	87	0.38	0.86	0.46	0.81	0.62 (0.53–0.71)	0.26 (0.09)	**<0.001**
Conduct disorder	27	13	6	21	7	99	0.46	0.83	0.22	0.93	0.64 (0.50–0.79)	0.19 (0.08)	**0.0073**
Oppositional defiant disorder^1^	30	20	10	20	10	93	0.50	0.82	0.33	0.90	0.66 (0.54–0.78)	0.24 (0.09)	**0.002**
Major depressive disorder	50	47	35	15	12	71	0.75	0.83	0.70	0.86	0.79 (0.71–0.86)	0.56 (0.09)	**<0.001**
Generalized anxiety disorder	34	50	33	1	17	82	0.66	0.99	0.97	0.83	0.82 (0.76–0.89)	0.69 (0.09)	**<0.001**
Separation-anxiety disorder	38	27	20	18	7	88	0.74	0.83	0.55	0.93	0.79 (0.69–0.88)	0.51 (0.09)	**<0.001**
Phobias	82	34	32	50	2	49	0.94	0.50	0.39	0.96	0.72 (0.6–0.78)	0.30 (0.07)	**<0.001**
Any externalised disorder^1^	50	40	24	26	16	67	0.60	0.72	0.48	0.81	0.66 (0.57–0.75)	0.30 (0.09)	**0.0002**
Any internalised disorder^2^	99	78	73	26	5	29	0.94	0.53	0.74	0.85	0.73 (0.66–0.80)	0.49 (0.085)	**<0.001**
Any disorder	106	104	92	14	12	15	0.89	0.52	0.87	0.56	0.70 (0.60–0.80)	0.41(0.09)	**<0.0001**

1 Includes attention-deficit/hyperactivity disorder, conduct disorder, and oppositional defiant disorder.

2 Includes major depressive disorder, generalised anxiety disorder, separation-anxiety disorder, and phobias.

The bold value means *p* < 0.05.

Looking at the Area under the curve, no values was below 0.50, but again values for internalized disorders were much higher than for externalized disorders, for whom the 95% CI could be lower than 0.50. Using “possible cases” slightly ameliorated the results.

### 3.2. Results of the community survey using *Dominic Interactive* in primary schools in Tianjin

Among the 1,479 children who successfully completed the DI survey, 498 (33.7%) were from the rural school, 763 (51.6%) from the suburban school, and 218 (14.7%) from the urban school; an average of 244 respondents were from each grade from grade 1 to grade 6. Respondents included 714 (48.3%) boys, 737 (49.8%) girls, and 28 (1.9%) who did not report their gender; their mean age was 9.1 (S.D. = 0.5) years, and the range in ages was from 6 to 13.

Among the 1,479 respondents who completed the family background questionnaire, 67.6% were the mothers and 27.9% were the fathers; 78.1% were living with their spouse, 13.7% were single parents, and 8.3% did not report their marital situation; 6.0% reported being well-off, 72.3% reported no financial difficulties, 13.1% reported financial difficulties, and 8.7% did not report their financial situation; 6.5% only completed primary school (or less), 43.3% had completed some secondary school, 31.4% had completed secondary school, 9.3% had a university degree, and 9.5% did not report their educational status.

At the last DI question asking to the child if “he or she enjoyed to play Xiaoming?” 91.75% of the children have answered “yes”: no gender difference was found but age difference showed up: those aged 8 years to 10 years enjoyed the most: 95.74% for the 9 years; the 6 years enjoyed it slightly less: 88.62% as the ageist: 88.19% for the 11 years, 89.60% for the 12 years (*p* = 0.031).

As shown in Table 3, 19% of the sample (24% of the girls and 13% of the boys) had one or more of the seven probable DI diagnoses, and 48% of the sample (56% of the girls and 37% of the boys) had one or more of the seven possible DI diagnoses. The prevalence of probable internalized disorders (ranging from 3.5% for major depressive disorders to 13.1% for phobia) was much higher than the prevalence of probable externalized disorders (from 1.0% for conduct disorder to 1.8% for oppositional defiant disorder); this pattern was also the case for possible DI disorders. Both probable and possible internalized disorders were more common in girls than boys and externalized disorders were more common in boys than girls, but these gender-based differences were only statistically significant for probable and possible phobias and for possible conduct disorders. The prevalence of situation-type phobias (elevators, hallways) was greatest in students from rural schools (30.7%), intermediate in students from semiurban schools (23.7%) and lowest in students from urban schools (17.4%)(Chi^2^ = 15.94, *p* < 0.0001). Environmental-type phobias (storm, heights) showed the opposite pattern: higher in urban and semiurban schools (45.8 and 45.5%, respectively) than in rural schools (36.3%; Chi^2^ = 6.61, *p* = 0.37).

**Table 3:** Prevalence per 100 respondents (95% CI) of probable or possible childhood mental disorders among 1,479 primary school children in Tianjin, China based on the results of the self-report *Dominic Interactive* video game.

Disorder	Probable cases				Possible cases (includes probable cases)			
	Girls and boys	Girls	Boys	p-value^1^	Girls and boys	Girls	Boys	*p*-value^1^
Attention deficit/ hyperactivity disorder	1.08% (0.62–1.75)	0.83% (0.30–1.79)	1.33% (0.64–2.43)	0.351	3.65% (2.75–4.74)	2.75% (1.69–4.22)	4.52% (3.15–6.25)	0.071
Conduct disorder	1.01% (0.57–1.67)	0.55% (0.15–1.40)	1.46% (0.73–2.60)	0.081	4.46% (3.47–5.64)	3.31% (2.13–4.88)	5.58% (4.05–7.46)	**0.034**
Oppositional defiant disorder	1.83% (1.21–2.65)	1.65% (0.86–2.87)	1.99% (1.12–3.26)	0.626	6.76% (5.53–8.16)	6.75% (5.03–8.83)	6.77% (5.08–8.81)	0.986
Major depressive disorder	3.52% (2.64–4.59)	4.27% (2.92–6.01)	2.79% (1.73–4.23)	0.122	8.45% (7.08–9.99)	10.06%(7.96–12.48)	6.91% (5.20–8.96)	0.29
Generalized anxiety disorder	2.23% (1.54–3.12)	2.48% (1.48–3.89)	1.99% (1.12–3.26)	0.526	6.42% (5.23–7.80)	7.16% (5.40–9.29)	5.71% (4.16–7.62)	0.255
Separation-anxiety disorder	5.48% (4.37–6.76)	5.79% (4.20–7.74)	5.18% (3.71–7.01)	0.609	12.17% (10.55–13.95)	13.36% (10.97–16.05)	11.02% (8.88–13.48)	0.169
Phobias	13.12% (11.44–14.94)	17.91% (15.18–20.89)	8.50% (6.61–10.72)	**<0.001**	41.24% (38.72–43.80)	51.65% (47.95–55.34)	31.21% (27.91–34.65)	**<0.001**
Any externalised disorder^2^	3.18% (2.34–4.20)	2.34% (1.37–3.72)	3.98% (2.70–5.64)	0.072	9.94% (8.46–11.58)	8.13% (6.24–10.36)	11.69% (9.48–14.20)	**0.022**
Any internalised disorder^3^	18.05% (16.12–20.11)	23.14% (20.12–26.38)	13.15% (10.82–15.77)	**<0.001**	46.52% (43.95–49.10)	56.20% (52.50–59.84)	37.18% (33.72–40.75)	**<0.001**
Any disorder	19.34% (17.35–21.44)	23.69% (20.64–26.96)	15.14% (12.65–17.90)	**<0.001**	48.48% (45.90–51.06)	56.47% (52.78–60.12)	40.77% (37.24–44.38)	**<0.001**

1 *p* values of Chi square analysis comparing proportion (i.e., prevalence per 100 respondents) in girls and boys.

2 Includes attention-deficit/hyperactivity disorder, conduct disorder, and oppositional defiant disorder.

3 Includes major depressive disorder, generalised anxiety disorder, separation-anxiety disorder, and phobias.

The bold value means *p* < 0.05.

As shown in Table 4, the correlation of the sum of the item scores for each of the seven diagnostic-specific subscales of the DI were all statistically significant. The strongest correlations occurred for the diagnoses that share items (i.e., items that are part of the scale score for both diagnoses): the strongest correlation (r = 0.82) is between the major depressive disorder subscale with the generalized anxiety disorder subscale, which share two items, followed by ADHD subscale (*r* = 0.72) which share four items; then three pairs of subscales that share two items have correlations ranging from 0.60 to 0.67. Despite the high correlations of subscale scores, comorbidity is relatively uncommon in this community-based population. Among the 1,479 school children, 77 (5.2%) met criteria for more than one probable DI diagnosis and 259 (17.5%) met criteria for more than one possible DI diagnosis; 28 (1.9%) met criteria for both a probable internalized disorder and a probable externalized disorder and 118 (8.0%) met criteria for both a possible internalized disorder and a possible externalized disorder.

**Table 4:** Correlation of the item scores for the seven diagnostic-specific subscales of the *Dominic Interactive* self-completion video game among 1,479 primary school children in Tianjin, China^1^ and number of shared items (DI questions) used in both of the DI diagnosis considered in each correlation coefficient.

		Phobia	Separation-anxiety disorder	Generalised anxiety disorder	Major depressive disorder	Oppositional defiant disorder	Conduct disorder	Attention deficit/hyperactivity disorder
Phobias	Correlation	1						
	Shared items	---						
Separation-anxiety disorder	Correlation	0.444	1					
	Shared items	0	---					
Generalized anxiety Disorder	Correlation	0.464	0.587	1				
	Shared items	0	0	---				
Major depressive disorder	Correlation	0.366	0.431	0.822	1			
	Shared items	0	0	2	---			
Oppositional defiant disorder	Correlation	0.291	0.341	0.603	0.670	1		
	Shared items	0	0	2	2	---		
Conduct disorder	Correlation	0.203	0.242	0.373	0.450	0.553	1	
	Shared items	0	0	0	0	1	---	
Attention deficit/hyperactivity disorder	Correlation	0.277	0.349	0.609	0.724	0.595	0.556	1
	Shared items	0	0	2	4	0	0	---

1 All presented correlation coefficients are statistically significant (*p* < 0.001).

Table 5 shows the results of multivariate logistic regression analyses of factors associated with the probable case or possible case DI classification of phobias – the most prevalent condition in the participating school children. After adjusting for all variables in the model, both probable and possible DI diagnoses of phobias were significantly more frequent in girls than in boys. Students from suburban and rural schools were significant more likely to have a probably DI diagnosis of phobias than students from urban schools, but this difference was not statistically significant when considering the possible DI diagnosis of phobias. Younger age was marginally associated with the possible DI diagnosis of phobias (*p* = 0.049) but not significantly associated with the probable DI diagnosis of phobia (*p* = 0.413). The marital status and educational level of the child’s guardian (i.e., the person who completed the household form) and the reported economic status of the household in which the child lived were not significantly associated with either the probable or possible DI diagnosis of phobia. A similar regression on the probable or possible diagnosis of any of the seven assessed disorders (Table 5) also found that girls were more likely than boys to have a probable or possible diagnosis and that students from suburban and rural schools were more likely than those from urban schools to have a probable diagnosis; but, unlike the results for phobia, age was unrelated to either a probable or a possible diagnosis and children from rural schools were more likely than those from urban schools to have a possible diagnosis.

**Table 5:** Logistic regression analysis of factors associated with the prevalence of possible and probable cases of phobia among 1,479 primary school children in Tianjin, China based on diagnostic result of the self-report *Dominic Interactive* video game.

Characteristic	Probable case of phobia		Possible case of phobia		Probable case of any of the 7 diagnoses		Possible case of any of the 7 diagnoses	
	Adjusted OR	*p*-value	Adjusted OR	*p*-value	Adjusted OR	*p*-value	Adjusted OR	*p*-value
	(95% CI)		(95% CI)		(95% CI)		(95% CI)	
Child’s age in years	0.96 (0.88–1.06)	0.413	0.94 (0.88–1.0)	**0.049**	0.94 (0.91–1.07)	0.695	0.95 (0.89–1.01)	0.128
Child’s gender								
Boy	1.00		1.00		1.00		1.00	
Girl	2.50 (1.78–3.53)	**<0.001**	2.49 (1.99–3.13)	**<0.001**	1.77 (1.34–2.35)	**<0.001**	2.00 (1.60–2.50)	**<0.001**
Child lives in single-parent household								
No	1.00		1.00		1.00		1.00	
Yes	1.38 (0.89–2.11)	0.147	1.06 (0.77–1.47)	0.703	1.41 (0.93–2.05)	0.07	1.13 (0.82–1.55)	0.45
Guardian’s report of household finances								
Financial difficulties	1.00		1.00		1.00		1.00	
No difficulties	0.90 (0.57–1.43)	0.658	0.92 (0.66–1.28)	0.612	0.93 (0.62–1.38)	0.717	1.02 (0.73–1.41)	0.912
Well-off	1.03 (0.48–1.19)	0.986	0.71 (0.41–1.24)	0.229	0.71 (0.35–1.44)	0.35	0.78 (0.46–1.33)	0.366
Guardian’s level of educational								
Primary school or less	1.00		1.00		1.00		1.00	
Some secondary school	0.80 (0.47–1.38)	0.428	0.81 (0.54–1.22)	0.324	1.12 (0.68–1.88)	0.641	0.90 (0.60–1.33)	0.592
Completed secondary school	0.71 (0.39–1.27)	0.245	0.79 (0.51–1.22)	0.282	1.19 (0.69–2.04)	0.525	0.84 (0.55–1.29)	0.432
Some college	0.61 (0.27–1.37)	0.229	0.71 (0.41–1.23)	0.227	0.70 (0.33–1.47)	0.351	0.72 (0.42–1.23)	0.226
Type of school								
Urban	1.00		1.00		1.00		1.00	
Suburban	2.67 (1.37–5.19)	**0.004**	1.24 (0.87–1.76)	0.237	1.87 (1.14–3.08)	**0.013**	1.29 (0.92–1.82)	0.140
Rural	2.63 (1.33–5.21)	**0.005**	1.41 (0.98–2.04)	0.067	1.92 (1.15–3.21)	**0.013**	1.50 (1.05–2.14)	**0.027**

The bold value means *p* < 0.05.

Based on the results from the 1,479 primary school children, the internal consistency of the 81 symptom-related items in the DI was excellent (Cronbach alpha = 0.93); the corresponding alpha values for the seven DI subscales (assessing the different types of mental health problems) were all acceptable (alpha = 0.87–0.64; Table 6).

**Table 6:** Validity measures on the 1,479 Children DI.

Scale/subscales	Number of items	Alpha	Chi squared	df	*p*-value	SRMR	Coefficient of determination
Complete scale	81	0.93	10,525.96	3,138	**<0.001**	0.062	0.941
Phobias	9	0.64	232.39	27	**<0.001**	0.051	0.679
Separation-anxiety disorder	8	0.65	125.96	20	**0.049**	0.041	0.671
Generalised anxiety disorder	15	0.80	869.89	90	**<0.001**	0.051	0.760
Major depressive disorder	20	0.87	1,299.21	170	**<0.001**	0.044	0.880
Oppositional defiant disorder	9	0.76	313.95	27	**<0.001**	0.053	0.790
Conduct disorder	14	0.74	504.98	77	**<0.001**	0.045	0.797
Attention deficit/ hyperactivity disorder	19	0.84	873.59	156	**<0.001**	0.046	0.856
Internalised disorder	46	0.90				0.061	0.919
Externalised disorder	41	0.89				0.063	0.903

The bold value means *p* < 0.05.

The Sem results were good: each SRMR (Standardized Root Mean Square Residual) was between 0 and 0.80, which is the upper limit recommended for a good fit (33). In addition, the coefficients of determination, which measure how well a statistical models predict outcomes were above 0.60 which has been recommended as the lower limit (34).

## 4. Discussion

The adapted Chinese version of DI was easy to administer, enjoyable for the students and acceptable to most parents. In the clinic-based validation study (which involved administering both the DI and the DAWBA) 82.2% of the parents of eligible children agreed to participate and 95% of these children completed the DI assessment; only 4 of the children reported discomfort completing the DI. In the school-based survey 96.7% of parents of eligible students agreed to participate and 72.1% of these children completed the DI; the lower rate of successful completion occurred because one parent in the urban school objected to the DI self-harm items resulting in the premature termination of the study in that school. Parental discomfort about the DI self-harm items – largely based on the incorrect belief that asking questions about self-harm increases the risk of self-harm – also occurred in a DI study in France where the slides had to be modified to make them more culturally acceptable (21). This recurring issue highlights the need to improve parental education about appropriate ways to discuss sensitive issues with their young children rather than trying to prevent their children’s exposure to such issues.

In the validation study the concordance, positive predictive values and negative predictive values of the DI probable and possible diagnoses with the “gold standard” DAWBA clinical diagnosis was better for the four internalized disorders (phobias, separation-anxiety disorder, generalized anxiety disorder, and major depressive disorder) than for the three externalized disorders (attention-deficit/hyperactivity disorder, conduct disorder, and oppositional-defiant disorder). Previous reports from France (21) suggest that self-reported externalized symptoms are acknowledged in a different manner than self-reported internalized symptoms: there is a normal distribution of self-reported internalizing symptoms, whereas the distribution of self-reported externalized symptoms is heavily right-skewed with many zero values (denial of all externalizing symptoms) and small numbers of symptoms in those who report any externalizing symptoms.

Indeed, in this comparison we are using two different instruments: DI and DAWBA but at the same time comparing different informants that are known to differ (35); in addition, the rate of externalized disorder was rather low for example as compared to what was obtained in the European surveys (24) where the average for externalized disorders was 7.8% that is the twice as high ranging from 10 to 3% in Turkey. It could be then, that the trend to self-report deviant behaviors were lower in societies less tolerant to these behaviors, whereas on the contrary, parents and teachers were more sensitive to them, increasing the discrepancy between evaluation.

Comparison of the results of the school DI survey in Tianjin to those of surveys in the European Union (EU) (24) found a similar combined prevalence of internalized disorders: 18.1% in China compared to the average prevalence of 18.4% in eight EU countries. However, the prevalence of specific internalized disorders varied: phobias were more common in the Chinese children (13.1% vs. 6.5%); separation-anxiety disorder was less common in Chinese children (5.5% vs. 11.4%); generalized anxiety disorder was less common in Chinese children (2.2% vs. 4.8%); and the prevalence of major depressive disorder was similar in Chinese and European children (3.5% vs. 3.8%). As we stated before, the combined prevalence of the three externalized disorders (based on the “probable” DI diagnosis) was much lower in Chinese children than European children (3.2% vs. 7.8%). This difference held true for each of the three externalized disorders: attention-deficit/hyperactivity disorder (1.1% vs. 4.5%), conduct disorder (1.1% vs. 3.5%), and oppositional-defiant disorder (1.8% vs. 4.2%).

The correlation of the seven subscales scores (Table 4) in this study are similar to the correlations reported in a Dutch sample (36); all seven subscale scores are significantly correlated with each other (Pearson’s correlation coefficient ranges from 0.20 to 0.82) with the highest correlations between the subscales that share items. The much higher prevalence of phobias in girls than boys (OR = 2.50) and the decreasing prevalence of phobias with increasing age has also been reported in European samples (24). However, unlike in the European samples, the prevalence of phobias in China was unrelated to the level of the educational level of the child’s guardian or to the marital status of the child’s guardian (that is, whether the child was living in a single-parent household). The higher prevalence of phobia and of any type of mental disorder in children from rural and suburban schools compared to the prevalence among children from urban schools in China has not been reported in European countries, but it is possible that differences in the degree of urbanicity between study samples from different European countries could partially explain reported differences in the prevalence of mental disorders in children from the different countries.

The internal consistency of the 81 clinical items in the DI was good (alpha = 0.93) and that of the items in the seven subscales was acceptable (alpha ranging from 0.64 to 0.87); the results are similar to those found in the Dutch sample (36). The results concerning the structural equation model for each of the seven scales were satisfactory as they were in the previous studies (37).

## 5. Limitations

There are several issues that need to be considered when interpreting these results.

1. We used DAWBA as our “gold standard” diagnosis. The DAWBA is based on detailed parent and teacher evaluations, revised by a trained clinician who judges the plausibility of the disorder on an algorithm plus a parent description; this is acceptable but is not equivalent to an in depth clinical interview of a clinician able to examine the child and to listen to the parents.
2. We compare at the same time the “informant” effect and the concordance effect; the children are more able to recognize their internalized disorders than their externalized disorders and consequently the concordances were much better for the first than for the second type of disorders. This implies that the DI should be used together with a parent/ teacher instrument such as the Strengths and Difficulties Questionnaire (SDQ) (38) to complete the evaluation, as we did in this survey and in the European surveys (39). As a matter of fact, this did not disqualify the DI, which is a unique instrument to get accurate information on internalized disorders on young children, since DI results could be reported together with other sources of information and may for some risk factors, represents the most sensible marker (24).
3. The national representativeness of our clinical sample of psychiatric patients and of our elementary school sample of community-dwelling children are unknown.

## 6. Conclusion

The Chinese version of *Dominic Interactive* proved easy to administer to children as young as 6 years of age both at home and in schools. It can be administered using a computer or tablet either individually or in groups. The short completion time (mean 18 min), non-threating method of obtaining diagnostically relevant information, coverage of the seven most common diagnoses in children, suitability for young children, and self-completion format (eliminating the potential complication of having adults administer or monitor the test) make the *Dominic Interactive* an ideal screening tool for identifying mental health problems in Chinese children.

Further work on the psychometric properties of the Chinese version of the DI is merited, but the cut points proposed by the author seem to fit for the Chinese children population. The DI questions about self-harm may need to be adjusted to make them more acceptable to Chinese parents.

Given increasing concerns about unaddressed mental health problems in China’s youth – particularly during the COVID-19 epidemic – the Chinese version of the DI could play an important role in screening large numbers of children and, thus, promoting the prevention and early treatment of children with disabling mental health conditions.

## Data availability statement

The raw data supporting the conclusions of this article will be made available by the authors, without undue reservation.

## Ethics statement

The survey was approved by the ethics committee of Tianjin Anding Hospital (Number 2019-01, Jan 9, 2019). Children could refuse any time. Anonymity was preserved. Written informed consent to participate in the study was provided and been signed by the participant’s legal guardian/next of the kin.

## Author contributions

VK-M participated in designing the study, conducted the analyses, and wrote the initial draft. HY participated in designing the study, coordinated the cleaning of the data, and participated to preparing the initial draft. GY diagnosed all the participants using DAWBA for the validation part of the study and organized the survey at the three schools. GX participating in designing the study. ML, PL, XS, and XH collected the DI data and entered and cleaned the data. LS interviewed cases from the pediatric outpatient department who participated in the study. MP extensively revised the initial draft of the manuscript and helped identify other components of the study that were subsequently reported in the manuscript. All authors contributed to the article and approved the submitted version.

## Funding

This research was funded by Tianjin Key Medical Discipline (Specialty) Construction Project (Number: TJYXZDXK-033A) and Tianjin Science and Technology Program (18ZXRHSY00100).

## Conflict of interest

The authors declare that the research was conducted in the absence of any commercial or financial relationships that could be construed as a potential conflict of interest.

## Publisher’s note

All claims expressed in this article are solely those of the authors and do not necessarily represent those of their affiliated organizations, or those of the publisher, the editors and the reviewers. Any product that may be evaluated in this article, or claim that may be made by its manufacturer, is not guaranteed or endorsed by the publisher.
